# Evaluation of Emulsification Techniques to Optimize the Properties of Chalcone Nanoemulsions for Antifungal Applications

**DOI:** 10.3390/ph17111442

**Published:** 2024-10-28

**Authors:** Joice Farias do Nascimento, Flavia Oliveira Monteiro da Silva Abreu, Taysse Holanda, Raquel Oliveira dos Santos Fontenelle, Júlio César Sousa Prado, Emmanuel Silva Marinho, Matheus Nunes da Rocha, Jesyka Macêdo Guedes, Bruno Coelho Cavalcanti, Wesley Lyeverton Correia Ribeiro, Márcia Machado Marinho, Helcio Silva dos Santos

**Affiliations:** 1 Postgraduate Program in Natural Sciences, Ceará State University, Fortaleza 60714-903, CE, Brazil; joice.nascimento@aluno.uece.br (J.F.d.N.); taysse.holanda@aluno.uece.br (T.H.); raquelbios@yahoo.com.br (R.O.d.S.F.); cesar.prado@aluno.uece.br (J.C.S.P.); emmanuel.marinho@uece.br (E.S.M.); matheusndarocha@gmail.com (M.N.d.R.); jesyka.mg@gmail.com (J.M.G.); 2 Department of Physiology and Pharmacology, Federal University of Ceará, Fortaleza 60430-160, CE, Brazil; bccavalcanti@gmail.com (B.C.C.); wesleylyeverton@yahoo.com.br (W.L.C.R.); 3 Center for Exact Sciences and Technology, Vale do Acaraú University, Sobral 62040-370, CE, Brazil; marinho.marcia@gmail.com

**Keywords:** drug delivery, nanotechnology, *Candida albicans*, homogenization, nanodroplets, molecular docking

## Abstract

**Background/Objectives:** Nanoemulsions (NEs) possess properties that enhance the solubility, bioavailability and therapeutic efficacy of drugs. Chalcones are compounds known for their antifungal properties. In this study, we evaluated different emulsification techniques to create alginate nanoemulsions containing chalcone (1E,4E)-1,5-bis (4-methoxyphenyl) penta-1,4-dien-3-one (DB4OCH_3_). Our goal was to develop an antifungal formulation targeting *Candida albicans* strains. **Methods:** Ultrasound and ultrasound combined with high-speed homogenization techniques were used to prepare alginate-stabilized nanoemulsions. Particle size, zeta potential and encapsulation efficiency were evaluated. Additionally, in vitro release studies were conducted. **Results:** The combined emulsification technique produced stable nanoparticles with high encapsulation efficiency and antifungal activity, with a minimum inhibitory concentration of 8.75 μg/mL for the nanoemulsions compared to 312 µg/mL for free DB4OCH_3_. NEs’ effectiveness can be attributed to their ability to form nanodroplets efficiently, facilitating the solubilization of the chalcone in the oily phase. The particle size varied between 195.70 ± 2.69 and 243.40 ± 4.49 nm, with an increase in chalcone concentration leading to larger particle sizes. The zeta potential showed values from −91.77 ± 5.58 to −76.90 ± 4.44 mV. The UHS-7 sample exhibited an encapsulation efficiency of 92.10% ± 0.77, with a controlled in vitro release of 83% after 34 h. Molecular docking simulations showed that the aromatic nature of DB4OCH_3_ resulted in the formation of apolar interactions with aromatic residues located in the active site of the TMK, as observed in their respective co-crystallized inhibitors, within an affinity energy range that enables optimum specificity of the ligand for these two pathways. Pharmacokinetic analyses indicated high passive cell permeability and low hepatic clearance, and phase I metabolism reduces its oral bioavailability and metabolic stability, suggesting a promising active ingredient as an oral drug with control of the daily oral dose administered. **Conclusions:** The combined nanoemulsification technique led to the formation of finely dispersed nanodroplets that favored the solubilization of the chalcone in the oil phase, which led to a better performance in the antifungal properties. DB4OCH_3_ shows promise as an oral drug with controlled dosing.

## 1. Introduction

Nanostructured drug delivery systems have received a great deal of attention due to their potential to protect the drug from degradation, allow prolonged release and increase the solubility, bioavailability and therapeutic efficacy of drugs [1,2]. Nanoemulsions (NEs) are known to increase the uniformity and stability of drug distribution [3,4,5,6]. NEs provide a strategic advantage in enhancing kinetic stability by encapsulating bioactive compounds like chalcones. Their small droplet size, ranging from 20–200 nm, reduces gravitational separation and improves overall stability [7]. For this reason, they are used in the development of drugs to improve their pharmacokinetic properties, such as chalcones [8,9,10].

Due to the increase in microbial resistance, which has caused significant hospitalizations and deaths in the world population over the last twenty years [11], it is necessary to research and produce new antimicrobial therapeutic products. Among the possible strategies are the development of new drugs through the molecular alteration of pre-existing drugs or the discovery of new substances with antimicrobial potential [12]. Chalcones are aromatic ketones with an α, β-unsaturated carbonyl system joining two aromatic rings. Belonging to the class of open-chain flavonoids, they can be synthesized easily [13], with antimicrobial properties [14], and include (1E,4E)-1,5-bis(4-methoxyphenyl)penta-1,4-dien-3-one (DB4OCH_3_) [15].

The production of nanoemulsions containing chalcones or chalcone-based compounds has been investigated to explore their antimicrobial and antifungal properties in recent years [16]. A study evaluated the efficacy of chalcone derivatives in NEs containing gum arabic and alginate against S. Minnesota, showing potent antimicrobial effects and no in vitro cytotoxicity [17]. Another study showed that 2-hydroxychalcone-loaded nanoemulsion has strong antifungal effects and is not toxic to Paracoccidioides brasiliensis and Paracoccidioides Lutzii [18]. The effects of alginate oil-in-water nanoemulsions containing chalcones were studied in previous research [19]. The chalcones created tiny droplets in the oil phase, allowing them to easily enter bacterial cells and display antimicrobial effects.

**Figure 1:**
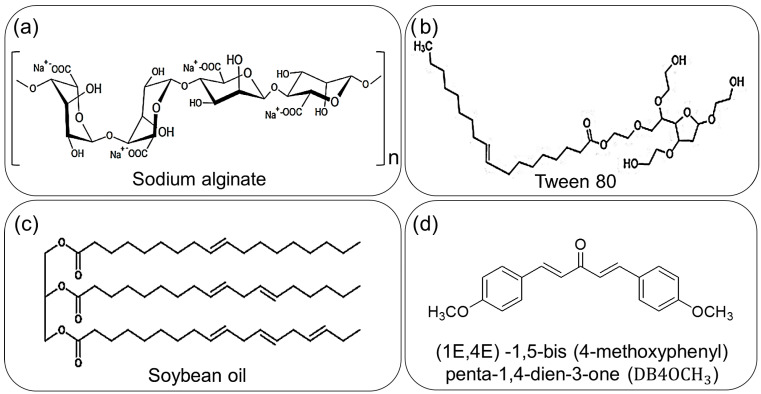
Representation of the components used in NEs; (**a**) the sodium alginate used as a stabilizer; (**b**) Tween 80 (T80) used as surfactant; (**c**) soybean oil used as co-surfactant and (**d**) the chalcone DB4OCH_3_, the synthetic compound with biological properties.

The stability and other properties of an NE are determined by its components. Besides the use of a commercial surfactant, the stability of an NE can be improved by incorporating co-surfactants and stabilizers. In a previous study, soybean oil, an inexpensive long-chain triglyceride (LCT), was employed to enhance the stability of the oily phase due to the drug’s hydrophobic nature and limited solubility in water. Figure 1 illustrates the chemical structures of the components employed in NE production.

This study aims to encapsulate the chalcone DB4OCH_3_ in a nanoemulsion with sodium alginate, comparing high-speed homogenization techniques combined with ultrasound to determine the best technique and chalcone concentration to enhance the antifungal efficacy of the chalcone DB4OCH_3_.

## 2. Results

### 2.1. Stability

**Figure 2:**
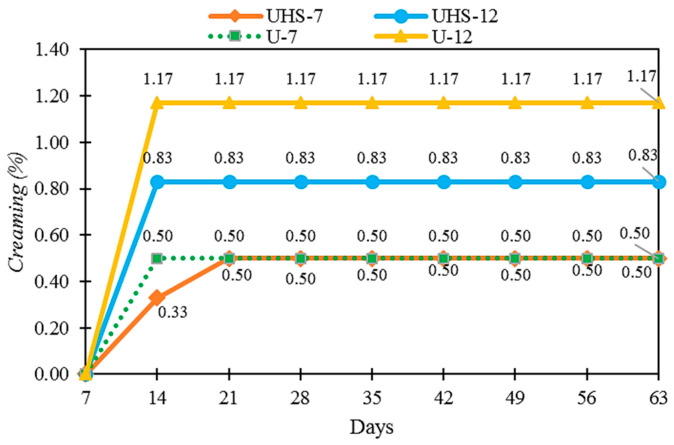
Creaming index (%) as a function of time (days) for samples produced by ultrasound and ultrastirrer (UHS-7 and UHS-12) and by ultrasound technique (U-7, U-12).

Samples processed using different techniques were proposed (UHS-7, UHS-12: high speed + ultrasound; U-7, U-12: ultrasound only). During the testing period, no sedimentation was observed in the formulations, indicating the stability and uniform homogenization of DB4OCH_3_. However, all the samples exhibited creaming (Figure 2). The samples UHS-12 and U-12 showed higher creaming, with values below 1.20%. The creaming index was lower with the combined techniques (CI = 0.83%) compared to the ultrasound technique (CI = 1.17%).

The samples with the same concentration, depending on the emulsification techniques (UHS-7 and U-7), exhibited different viscosity values. When the nanoemulsions (NEs) were diluted to 0.1%, the following viscosity results were obtained: 5.2 dL/g for sample UHS-7, 5.5 dL/g for sample UHS-12, 4.1 dL/g for sample U-7 and 4.2 dL/g for sample U-12. The samples developed with the high-speed technique combined with ultrasound showed higher viscosity, whereas those produced solely with ultrasound exhibited lower viscosity. Ultrasonic cavitation induces high shear force and temperature, resulting in the degradation of polysaccharides and reduction in viscosity.

### 2.2. Morphology of Nanoemulsions

**Figure 3:**
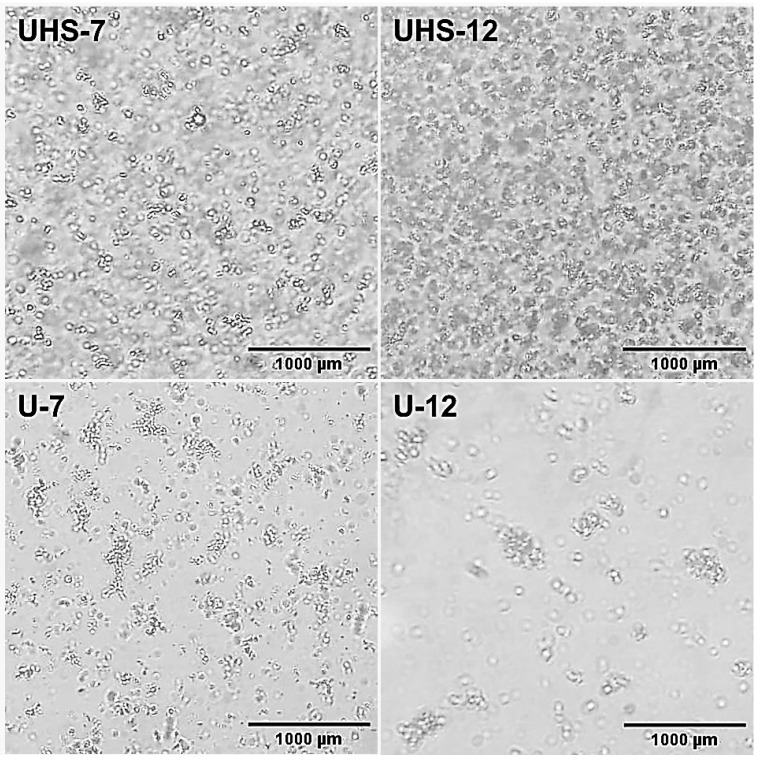
Micrographs of samples UHS-7, UHS-12, U-7 and U-12.

Figure 3 exhibits the optical microscopy of the agglomerated oil droplets surrounding the continuous phase, with micrographs taken at 400× magnification. Domains were observed in all samples, with a tendency to form aggregates. The homogeneity of UHS-7 was higher, featuring a greater quantity of dispersed oil droplets. UHS-12, on the other hand, had more droplets that tended to aggregate. Agglomerated droplets were observed in both U-7 and U-12, with U-7 exhibiting smaller domains than U-12.

**Figure 4:**
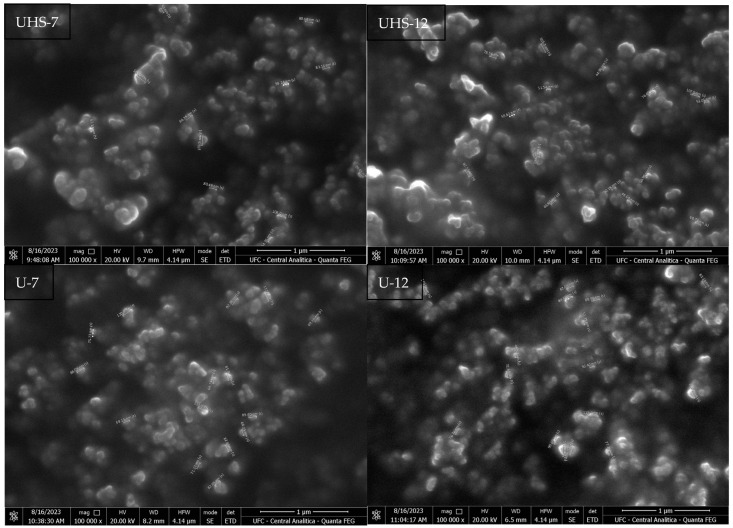
Scanning electron microscopy of samples UHS-7, UHS-12, U-7 and U-12.

SEM analysis showed that the average size of the dried film NEs was 72.50 ± 16.21 and 73.83 ± 20.91 nm for the UHS-7 and UHS-12 samples. In comparison, the NEs in U-7 and U-12 showed larger average sizes, measuring 80.62 ± 15.67 nm and 84.76 ± 12.44 nm, respectively (Figure 4). All the NEs fall within the 1-100 nm range. The larger size of the U1 and U2 samples might be a result of less efficient emulsification using solely the ultrasound technique, potentially impacting the stability and bioavailability of the NEs.

### 2.3. Particle Size, Zeta Potential and Polydispersity Index

The data in Table 1 show that the particle sizes range from 195.70 ± 2.69 to 243.40 ± 4.49 nm. All the formulations have sizes below 250 nm. The slightly larger size in samples U-7 and U-12 may be attributed to less efficient emulsification with ultrasound alone, which could affect the stability and bioavailability of the NEs.

The PdI of our samples ranged from 0.492 ± 0.01 to 0.525 ± 0.01, showing a moderate degree of polydispersity, while only PdI values below 0.3 suggest a monodisperse system. The zeta potential exhibited highly negative values, indicating strong electrostatic stability ranging from −91.77 ± 5.58 to −76.90 ± 4.44 mV.

The samples that exhibited the highest encapsulation efficiency were UHS-7 (92.1%) and UHS-12 (91.7%), while the samples produced with ultrasound had efficiencies of 74.0% (U-12) and 39.4% (U-7). This demonstrates that encapsulation efficiency is directly related to the homogenization technique used, with better results observed when combining the high-speed homogenizer with ultrasound.

### 2.4. Antifungal Properties of Nanoemulsions

The minimum inhibitory concentration (MIC) of free DB4OCH_3_ and the nanoemulsions (NEs) against C. *albicans* are presented in Table 2. Samples UHS-7 and UHS-12 demonstrated antifungal activity, while samples U-7 and U-12 did not show this activity.

The nanoemulsions (NEs) UHS-7 and UHS-12 exhibited excellent antifungal activity against the *C. albicans* strains. UHS-7 proved to be the most effective, showing an MIC of 8.75 μg/mL for LABMIC (0104) and LABMIC (0128), and an MIC of 17.5 μg/mL for ATCC (90028), LABMIC (0102) and LABMIC (0105). In NE UHS-12, LABMIC (0105) had an MIC of 37.5, whereas the strains ATCC (90028), LABMIC (0102), LABMIC (0104) and LABMIC (0128) all had an MIC of 75 μg/mL. The NEs displayed greater effectiveness (8.75–75 μg/mL) compared to free DB4OCH_3_, which had a significantly higher MIC (312–625 μg/mL). UHS-7 exhibited an MIC approximately 35 times lower, while UHS-12 showed an MIC around 18 times lower than the free compound.

### 2.5. Cytotoxicity

Table 3 presents the cytotoxicity values for DB4OCH_3_, UHS-7, UHS-12, U-7 and U-12. DB4OCH_3_ showed an IC_50_ greater than 150 µg/mL, indicating toxicological safety in the MTT assay. The formulations UHS-7, UHS-12, U-7 and U-12 exhibited IC_50_ values lower than DB4OCH_3_, but still higher than the positive control. Additionally, none of the samples induced rupture of the erythrocyte plasma membranes, indicating the absence of hemolytic events in all treatments.

### 2.6. In Vitro Release Kinetic Profile

**Figure 5:**
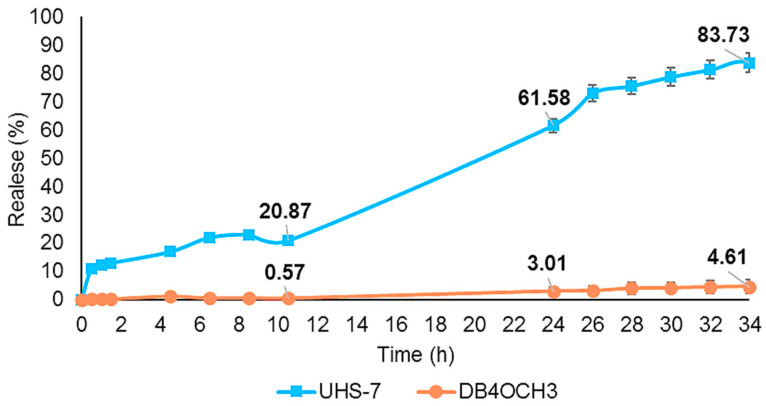
In vitro controlled release profile of UHS-7 and free chalcone DB4OCH_3_.

The in vitro release profile of the NEs and the free sample diluted in DMSO, conducted at pH 7.0, is shown in Figure 5. After 10 h, UHS-7 released 20% of DB4OCH_3_, increasing to 61.5% after 24 h and approximately 83.7% after 34 h. Conversely, according to the analysis, free DB4OCH_3_ exhibited a release of less than 1% after 10 h, 3% after 24 h and 4.6% after 34%. The restricted release implies that DB4OCH_3_ has low solubility in water-based mediums. The release of the DB4OCH_3_ compound is greatly enhanced when using UHS-7 due to the controlled and prolonged release mechanism provided by the nanoemulsions.

Table 4 presents the kinetic constant and the correlation coefficient for the studied kinetic models. The Hixson–Crowell model provided the best fit for NE UHS-7, and the zero-order model was the best fit for DB4OCH_3_, with a high correlation coefficient (R^2^) in both cases. Figure 5 shows a controlled in vitro release of NE UHS-7 and the free chalcone DB4OCH_3_ after 34 h of analysis.

## 3. Discussion

### 3.1. Stability

Cream formation occurs when oil droplets separate and move towards the surface, forming a layer. This phenomenon is related to the density difference between the dispersed and continuous phases, influenced by the force of gravity. The higher oil phase content in the UHS-12 and U-12 samples is explained by the increased amount of chalcone, which justifies the greater cream formation. This process occurs due to the oil phase, where gravity acts on the detached droplet, causing the less dense layer to rise to the surface [19]. The lower cream formation in the combined techniques suggests a more efficient homogenization compared to the ultrasonic technique.

Viscosity tends to increase with higher oil phase concentration due to the increased interfacial tension with the aqueous phase [20]. However, the difference in viscosity values between samples with the same concentration but produced using different emulsification techniques suggests that the production technique directly influences viscosity. Samples produced with ultrasound exhibit lower viscosity because ultrasonic cavitation induces high shear force and temperature, resulting in the degradation of polysaccharides. This breakdown of polysaccharide bonds reduces viscosity, as these bonds form a three-dimensional network that lowers the emulsion viscosity [21]. Conversely, higher viscosity contributes to emulsion stability by kinetically stabilizing the particles and reducing the chances of collision, coalescence and cream formation. Thus, NEs with higher viscosity, produced by the combined ultrasound and high-speed homogenization techniques, are more stable and advantageous [22].

### 3.2. Morphology of Nanoemulsions 

The morphological analysis showed that the UHS-7 and UHS-12 samples have greater homogeneity and less flocculation, with UHS-7 being the most homogeneous due to the higher number of dispersed oil droplets. Samples U-7 and U-12, although with smaller domains in U-7, showed a tendency to flocculate, while U-12 exhibited larger domains, possibly due to the lower number of oil droplets. The higher concentration of DB4OCH_3_ caused the oil droplets to increase in size, explaining the difference in behavior between the UHS-7 < UHS-12 and U-7 < U-12 samples.

SEM analysis of the dried films indicated that the shape and size of the NEs are within the expected range for nanoemulsions (1–100 nm), with the higher concentration of DB4OCH_3_ contributing to the increase in the average particle size. This behavior confirms the observations that higher concentrations of the oil phase lead to an increase in oil droplets size [23,24].

### 3.3. Particle Size, Zeta Potential and Polydispersity Index 

Particle sizes below 250 nm indicate good kinetic stability, due to the excellent zeta potential of the formulations, which favors particle stability [25]. The particle sizes of the formulations UHS-7, UHS-12 and U-7 were approximately 200 nm, allowing effective interaction with the microorganism cell walls [17]. The discrepancy between the particle size values obtained from the zeta sizer and SEM can be explained by the presence of solvent molecules and surface layers in suspension, leading to a larger particle size in the zeta sizer compared to the SEM technique, which involves solvent evaporation [26].

The polydispersity index suggests moderate particle homogeneity, with values lower than 0.3 being preferable for greater uniformity [25]. This implies the presence of a broader range of particle sizes. However, nanoemulsions showed other stability indicators, including droplet size, zeta potential above ±30 mV, optical clarity, consistent viscosity and resistance to phase separation, supporting their stable nature. Particularly, the negative values are characteristic of excellent electronic stabilization and have been related to the carboxyl groups of the ALG, which coat the droplets [27].

Additionally, the nanometric size of the droplets and the controlled release profile make these formulations suitable for topical use, with the ideal average size range between 100 and 350 nm [28].

Encapsulation efficiency is influenced by the production technique. The combined use of high-speed homogenization with ultrasound resulted in significantly higher encapsulation efficiency. Previous studies support this observation, where Yu and Huang [29] encapsulated curcumin using the ultrasound technique and achieved an efficiency of 76.30% ± 1.0, while Ali et al. [30] reached 68.05% ± 1.2 with the high-speed technique. The combination of these techniques promotes greater interaction between the formulation components, resulting in a more uniform distribution of DB4OCH_3_ and minimizing chalcone loss.

### 3.4. Antifungal Properties of Nanoemulsions

When adjusting the production technique and chalcone concentration in four different formulations, we discovered that the most effective way to produce DB4OCH_3_-loaded sodium alginate nanoemulsions is by combining high-speed homogenization with ultrasound. Increasing the content of DB4OCH_3_ in the nanoemulsion did not lead to improved antifungal activity against *C. albicans*. The increased content of DB4OCH_3_ in the oily phase resulted in larger droplets, potentially hindering effective interaction with microorganism cell walls [17]. Tiny, dispersed droplets containing lower amounts of DB4OCH_3_ exhibited antifungal properties against *C. albicans*. This suggests that the homogenization technique has a direct impact on the antifungal efficacy of the nanoemulsions.

Previous studies have shown that nanoemulsions of cinnamaldehyde inhibited *C. albicans* with an MIC of 19.92 mg/L [31] and polymeric nanoemulsions containing chalcone derivatives inhibited *C. albicans* with an MIC ranging from 0.625 to 0.175 mg/mL [27]. Comparing these studies with the results obtained by our team, it was concluded that this study produced better results, with the NEs UHS-7 and UHS-12 showing much lower MICs, especially UHS-7, which demonstrated greater efficacy at the lowest concentration tested.

The antifungal activity of chalcones and their derivatives is due to the presence of α, β-unsaturated carbonyl groups, which enhance their activity [32]. The observed inhibition may be related to interference with the biosynthesis of plasma membrane and cell wall components, such as ergosterol, β(1 → 3) glucan and chitin, causing cell leakage and microorganism death [33]. Additionally, using NEs as a vehicle for DB4OCH_3_ appears to have significantly enhanced antifungal activity, allowing lower concentrations of chalcone to have a greater inhibitory effect than the free form of the compound.

### 3.5. Cytotoxicity

Cytotoxicity measures a substance’s potential to cause cellular damage or death, evaluated through various methods such as the MTT assay. The observed difference in IC50 values between the nanoformulations and free DB4OCH_3_ may be related to the size of the nanoparticles and the increased specific surface area, which facilitates interaction with cellular components, as reported by Buzea, Pacheco and Robbie [34] and Egbuna et al. [35].

These changes can influence the cytotoxicity pattern, as indicated by Pushpalatha, Selvamuthukumar and Kilimozhi [36]. The need to optimize the nanoparticulated system to reduce its toxicity compared to the free compound or to improve the cell culture conditions in the MTT assay is a point suggested by the results.

Despite the observed cytotoxicity, the absence of hemolytic events indicates that the formulations are safe for use, as no rupture of red blood cells was identified. This finding is consistent with previous studies by Mann et al. [37], who also reported similar hemolytic results with chalcone derivatives. Additionally, Zhang et al. [38] suggested that chalcones have the ability to inhibit hemolytic enzymes produced by *Staphylococcus aureus*, which may contribute to the safety of the compounds.

### 3.6. In Vitro Release Kinetic Profile 

The results demonstrate that nanoemulsion (NE) acts as an effective vehicle to improve drug solubility, facilitating its homogeneous dispersion and significantly increasing its drug release [39]. This is evidenced by the in vitro release profile of UHS-7, which released a significantly higher amount of the drug, with 83% of release after 34 h compared to free DB4OCH_3_, which had only 4% of release in the same period, due to their low solubility. The use of UHS-7 greatly enhances the release of the DB4OCH_3_ compound by leveraging the controlled and prolonged release mechanism offered by nanoemulsions.

NEs have the advantage of providing a controlled and prolonged release of active substances, which is particularly useful for transdermal systems, osmotic systems and drugs with low solubility [40]. The kinetic analysis confirmed that the Hixson–Crowell model was the most suitable for describing the release profile of the NEs, showing that the dissolution rate of DB4OCH_3_ was dependent on the surface area of the particles. The low droplet size of the NEs provided a large surface area, which enhanced the dissolution rate of the active ingredient, releasing the drug consistently and in a controlled manner over time.

### 3.7. Molecular Docking Simulations

#### 3.7.1. Binding Mode of the DB4OCH_3_ with Thymidylate Kinase (ID: 4QGG)

In the investigation of the theoretical antimicrobial mechanism of DB4OCH_3_, it was possible to observe that the compound has an excellent affinity energy (EA) for the enzyme Thymidylate Kinase (TMK), with a calculated value of −6.767 kcal/mol, although it showed a lower modulus when compared to the co-crystallized ligand 32C, with a calculated value of −7.67 kcal/mol, and the comparative antimicrobial oxaciclin (OXA), with an EA value of −7.233 kcal/mol (Table 5), indicating that the ligands have good specificity for the target binding site. In addition, the compounds performed in the molecular docking simulations within an ideal statistical threshold, formed by RMSD values of less than 2.0 Å [9], allowing the reproducibility of the simulation model involving TMK.

**Figure 6:**
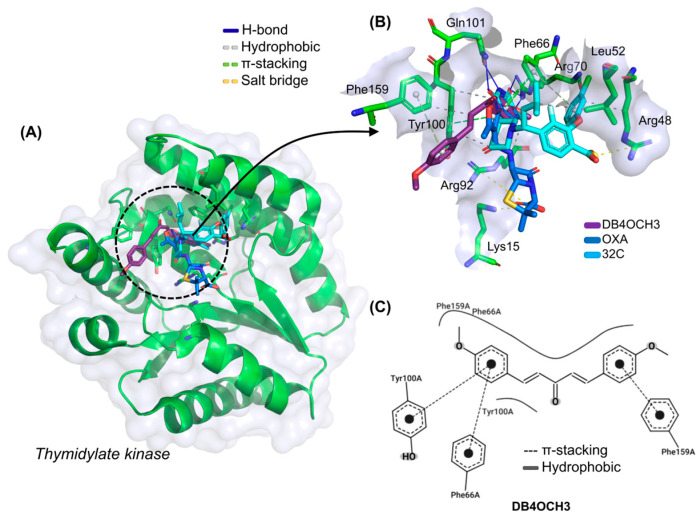
(**A**) Three-dimensional representation of the docking of the ligands DB4OCH_3_ (purple), OXA (blue) and the co-crystallized inhibitor 32C at the inhibition site of the TMK enzyme, (**B**) three-dimensional representation of the ligand–receptor interactions and (**C**) 2D map of the structural contribution of DB4OCH_3_ in the interactions with the residues of the TMK binding site.

At the end of the cycle of independent molecular docking simulations, it was possible to see that DB4OCH_3_ complexed with TMK in the same binding site as the native inhibitor and the OXA control (Figure 6A), especially by forming common interactions with amino acid residues that interact with 32C. The interactions in common include apolar interactions with the Phe66 and Tyr100 residues and an H-bond interaction with the Arg70 residue, residues strongly related to TMK inhibition (Figure 6B). In addition, the OXA control also formed hydrophobic interactions with the Phe66 residue.

In the analysis of the structural contributions in the ligand–receptor interactions of DB4OCH_3_ with the TMK enzyme, it was possible to observe that the compound formed hydrophobic interactions of the π-stacking type with the aromatic portion of the Phe66 and Tyr100 residues, with a strong contribution from one of its methoxylated aromatic rings (Figure 6C), as well as forming an H-bond interaction with the polar portion of Arg70 via a methoxy group from the other aromatic ring (H3C-O···H-NH), where the donor–acceptor distance of around 2.86 Å characterizes an interaction of moderate strength.

#### 3.7.2. Binding Mode of the DB4OCH_3_ with DNA Gyrase B (ID: 6F86)

When evaluating the molecular docking simulations of DB4OCH_3_ against the DNA Gyrase B enzyme, it was possible to observe that the compound presented a better EA for the target, with a calculated value of −6.101 kcal/mol, when compared to the co-crystallized inhibitor CWW, with an EA value of −5.035 kcal/mol (Table 5). The simulations involving DB4OCH_3_ performed within a lower root mean square deviation (0.563 Å), although all the compounds showed favorable values (RMSD < 2.0 Å).

**Figure 7:**
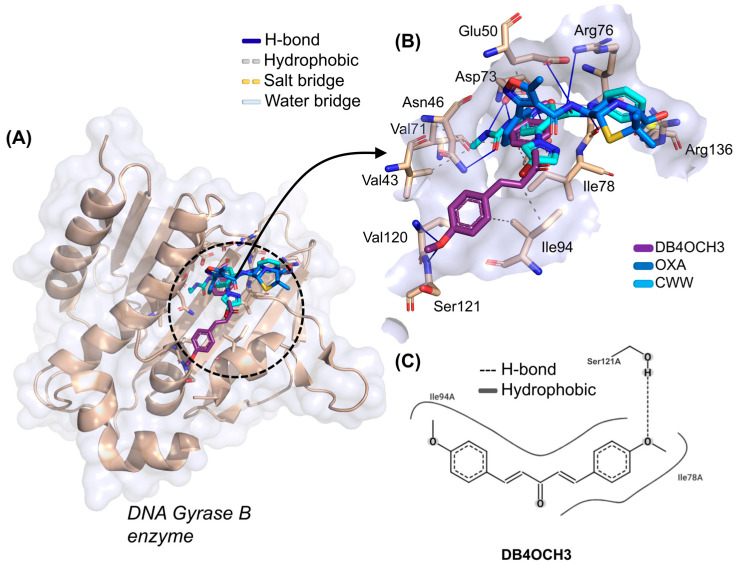
(**A**) Three-dimensional representation of the docking of the ligands DB4OCH_3_ (purple), OXA (blue) and the co-crystallized inhibitor CWW at the inhibition site of the DNA Gyrase B, (**B**) three-dimensional representation of the ligand–receptor interactions and (**C**) 2D map of the structural contribution of DB4OCH_3_ in the interactions with the residues of the DNA Gyrase B binding site.

At the end of the cycle of simulations, it was possible to observe that the best fitting pose for DB4OCH_3_ is in the vicinity of the Gyrase B catalytic site, where the co-crystallized inhibitor CWW and the control OXA are located (Figure 7A). Here, it was possible to notice that the substance formed some interactions in common with the CWW inhibitor, which include a hydrophobic interaction with the apolar side chain of the Ile78 residue and an H-bond interaction with the polar portion of the Thr165 residue (Figure 7B). However, these are residues unrelated to the catalytic activity of Gyrase B, which include the polar residues of Asn46, Asp73, Gly77, Arg136 and Pro79.

Analysis of the structural contributions reveals that the methoxy (-OCH_3_) portion of DB4OCH_3_ formed an H-bond interaction with the H-bond donor hydroxyl (-OH) of the side chain of the Ser121 residue (H3C-O···H-O). However, the co-crystallized inhibitor shows a higher number of H-bond interactions, including a strong interaction with the Gly77 residue with a donor–acceptor distance in the order of 1.81 Å, when compared to the stronger interaction of DB4OCH_3_ (2.63 Å).

### 3.8. MPO-Based DMPK Prediction

#### 3.8.1. MPO Analysis

Analysis of the alignment between the polar surface and the molecular lipophilicity potential (MLP) is crucial to understanding accessibility to hydrophobic and lipophilic solvents. In addition, this analysis provides insights into diffusion across various biological barriers, such as the lipid bilayer of cells. Compounds with MW > 200 g/mol that are within a range of lipophilicity at physiological pH (logD7.4) calculated between 0.0–3.0 showed greater cell permeability in Caco-2 cells in assays. In addition, more polar compounds (TPSA > 20 Å^2^), within the low toxicity lipophilicity threshold, showed greater permeability in MDCK cells. These cell lines are widely used in assays to estimate the oral absorption and metabolic stability of drug candidates.

**Figure 8:**
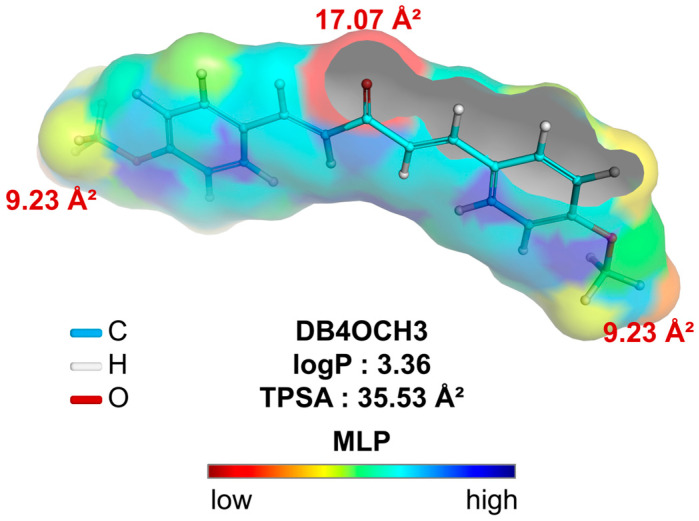
Molecular lipophilicity potential (MLP) map plotted to analyze the relationship between apparent lipophilicity (logP) and topological polar surface area (TPSA), where the color spectrum ranges from red (hydrophilic fragments) to blue (hydrophobic fragments).

In a topological analysis, the inductive electron-withdrawing effect of the carbonyl of the aliphatic system results in a decrease in the electronic density of the aromatic rings, forming an essentially lipophilic molecular surface (green to blue color spectra). In addition, the polar surface formed by the OCH3 groups (yellow color spectra), with surface areas equal to 9.23 Å^2^, and the C=O group (red color spectra), with a polar surface area equal to 17.07 Å^2^, result in a calculated overall polarity of 35.53 Å^2^ (Table 6) and, consequently, a balance between lipophilicity and solubility, with a logP value calculated at 3.36 (Figure 8).

By calculating the molecular descriptors, it was possible to observe that the compound basically follows the medicinal chemistry trends of the Pfizer, Inc. (Brooklyn, NY, USA) classification system (Figure 6A), including lipophilicity (logP < 5), molecular weight (MW < 360 g/mol), polarity (TPSA > 20 Å^2^) and basicity (non-ionizable), resulting in a multiparametric optimization (MPO) score of around 4.90 (on a scale of 0 to 6), indicating favorable pharmacokinetic viability (Table 6).

#### 3.8.2. PAMPA Prediction

Compounds that reside in a favorable physicochemical space (MPO > 4.0) have a higher lipophilicity–metabolism efficiency, which ensures alignment between in vitro pharmacokinetics descriptors based on Parallel Artificial Membrane Permeability Assay (PAMPA) properties, which include high passive cell permeability (Papp, A → B > 5 × 10⁻⁶ cm/s), reduced P-gp-mediated efflux and low hepatic clearance (CLMicro < 8 mL/min/kg). 

**Figure 9:**
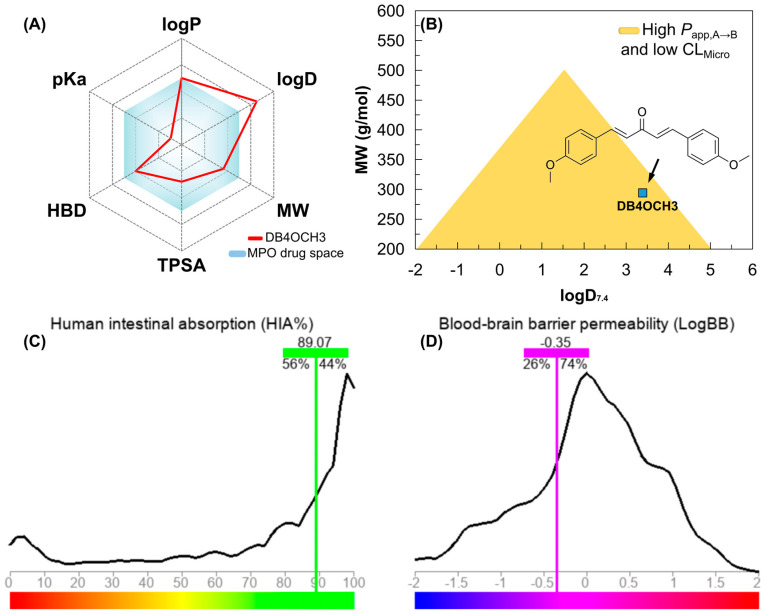
(**A**) multiparameter optimization (MPO) radar for estimating the pharmacokinetic and pharmacodynamic viability of DB4OCH_3_, (**B**) alignment between lipophilicity at physiological pH (logD7.4) and molecular weight (MW) for estimating the Papp, A → B and CLMicro profile and predicting (**C**) the percentage of human intestinal absorption (HIA) and (**D**) the coefficient of permeability at the blood–brain barrier (logBB).

With the MPO analyses, it was possible to observe that DB4OCH_3_ occupies a physicochemical space formed by compounds with MW between 200–300 g/mol and buffer lipophilicity (logD7.4) lower than 4.0, indicating a high probability of showing an alignment between high Papp, A → B and low CLMicro (Figure 9B). Corroborating this, a Papp, A → B in the order of 10⁻^5^ cm/s was predicted for the Caco-2 (intestine-like) and MDCK (BBB-like) cell lines, within the consensus test involving deep learning-based predictive tools (Table 7). When aligned, these descriptors are indicative of high cell viability and uptake in different physiological compartments, enhanced by low hepatic clearance (CLMicro < 15 mL/min/kg) and P-gp inhibition (Table 7). In parallel, DB4OCH_3_ showed similarity with at least 56% of the compounds deposited in the ADMET–LMC database with high human intestinal absorption (HIA), estimating a relative value of 89.07% (Figure 9C), including 26% of the compounds permeable to the blood–brain barrier (BBB), estimating a permeability coefficient (logBB) in the order of −0.35 (Figure 9D). This attribute is related to the relatively balanced molecular surface between the apolar surface (aromatic rings) and the polar surface (H-bond acceptor groups). In addition, it was noted that moderate lipophilicity can shift the distribution equilibrium of DB4OCH_3_. Thus, the predicted VD values can vary between 0.5 and 1.8 L/kg, indicating that the compound is widely distributed in the blood plasma.

#### 3.8.3. Liver and hERG Toxicity Prediction

**Figure 10:**
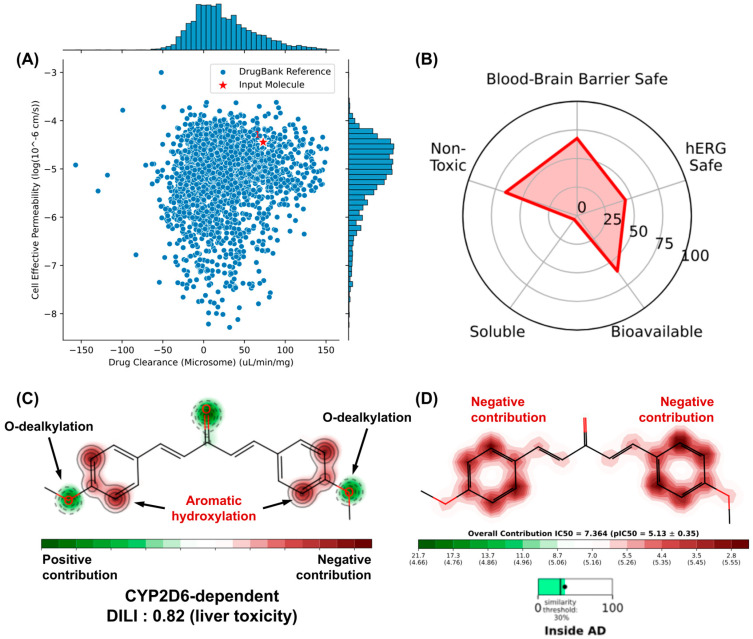
(**A**) similarity test with compounds with optimized cell permeability and hepatic clearance properties deposited in the DrugBank^®^ database, (**B**) prediction of toxicity endpoints and analysis of the structural contributions of DB4OCH_3_ to the model of (**C**) metabolism site and (**D**) hERG channel inhibition.

Machine learning model-dependent toxicity predictions are very useful in estimating the binding of small molecules to CYP450 metabolizing isoforms and K⁺ hERG transport channels, as indicative of toxicity endpoints associated with hepatotoxicity and cardiotoxicity, respectively. The similarity test with compounds deposited in the DrugBank^®^ database showed that DB4OCH_3_ occupies a physicochemical space formed by substances with high effective cell permeability and low hepatic clearance (Figure 10A), while the pharmacokinetic attribute endpoints estimated a considerable percentage of the compound to be a possible hERG channel inhibitor, which could result in cardiotoxic damage (Figure 10B).

An analysis of the metabolism site of DB4OCH_3_ showed that the compound has two OCH3 groups that are highly sensitive to phase I biotransformation, mostly dependent on the CYP2D6 isoform in the human liver (Figure 10C). Although this has a positive structural contribution (safe), the formation of secondary metabolites by O-dealkylation can affect the oral bioavailability of the substance (Figure 10B). On the other hand, the compound’s aromatic rings are susceptible to hydroxylation and can form epoxide intermediates that are reactive against proteins and DNA, corroborating the 0.82 probability of the compound inducing liver damage (DILI descriptor). In addition, these aromatic centers contribute to the formation of hydrophobic surfaces capable of binding to the hydrophobic cavities of hERG channels, where inhibition of this pathway leads to a cardiotoxic response, such as cardiac arrhythmia (Figure 10D).

## 4. Materials and Methods

### 4.1. Materials

The sodium alginate (DINÂMICA), surfactant Tween 80^®^ (VETEC), commercial soybean oil and synthetic chalcone 1,5-bis (4-methoxyphenyl) penta-1,4-dien-3-one (DB4OCH_3_) were provided by the Natural Products Chemistry Laboratory of the State University of Ceará. For the *Candida albicans* (*C. albicans*) test, Sabouraud Dextrose Agar (SDA), Roswell Park Memorial Institute medium (RPMI 1640), L-glutamine and morpholinopropanesulfonic acid (MOPS) were used.

### 4.2. Production of Polymeric Nanoemulsions Carrying Chalcones

The NEs were produced by the high-energy technique, using a rotor-stator (Ultra stirrer high-speed homogenizer model ULTRA380) and ultrasound (Ultronique QR500 Ultrasonic Tip Sonicator, Indaiatuba, Brazil). Table 8 shows the techniques and concentrations used.

ANOVA was used to evaluate the properties of the formulations, selecting particle size, viscosity and zeta potential as the dependent variables. The independent variables were polysaccharide matrix and oil phase composition. The factors related to the independent variables and their high (HL) and low (LL) levels were defined as follows:

- Factor A: DB4OCH_3_ Concentration: LL 0.07 mg/mL (−); HL 1.20 mg/mL (+);
- Factor B: Technique: HL Ultrasound and high-speed (+); LL: Ultrasound (−).

### 4.3. High-Speed Homogenization Combined with Ultrasound

Two nanoemulsions were created, with varying chalcone concentrations of 0.07 mg/mL and 1.20 mg/mL. The oil phase ratios of the DMSO/surfactant/vegetable oil (2/2/1) remained constant. The initial step involved dissolving the chalcone (7 mg or 12 mg) in 2000 μL of DMSO, followed by adding the oil phase (2000 μL of Tween 80 + 1000 μL of vegetable oil). The mixture underwent continuous homogenization for 1 min using an Eco-sonics model QR350 sonicator operating at an ultrasonic frequency of 20 kHz and a power of 440 W. Afterwards, the mixture was gradually added to the volume of ALG (1% *w*/*v*) using a syringe. An Ultra stirrer homogenizer model ULTRA380 was used to mechanically homogenize the formulation at 22,000 rpm for three minutes at room temperature (25 °C).

### 4.4. Ultrasonic Tip Sonicator

We created two separate nanoemulsions while maintaining a constant ratio of DMSO/surfactant/vegetable oil (2/2/1), with the chalcone content varying by 0.07 mg/mL and 1.20 mg/mL. The chalcone (7 mg or 120 mg) was initially dissolved in 2000 μL of DMSO, and then the oil phase (2000 μL of DMSO + 2000 μL of Tween 80 + 1000 μL of vegetable oil) was added. The mixture was continuously homogenized for 1 min using an Eco-sonics model QR350 sonicator (Indaiatuba, Brazil), operating at an ultrasonic frequency of 20 kHz and a power of 440 W, along with half of the volume of a 1% *w*/*v* ALG solution. The remaining 1% *w*/*v* ALG solution was added to the mixture and stirred for an additional 2 min at room temperature (25 °C).

### 4.5. Preparation of the Inoculum for Antifungal Sensitivity Tests

The inoculum was prepared from cells grown on Sabouraud Dextrose Agar (SDA) and incubated at 35 °C for 24 h. The yeast colonies were transferred to tubes containing sterile saline solution to obtain suspensions with turbidity equivalent to 0.5 on the McFarland scale (106 CFU/mL). These suspensions were then diluted 1:2000 with Roswell Park Memorial Institute medium (RPMI 1640), supplemented with L-glutamine, with pH adjusted to 7.4 with morpholinopropanesulfonic acid (MOPS) 0.165 mol L^−1^ (Sigma Chemical Co., St. Louis, MO, USA), according to the instructions of the Clinical and Laboratory Standards Institute (CLSI) M27-A3 2008, ATCC 90028 (*C. albicans*), LABMIC 0102 (*C. albicans*).

### 4.6. Stability Study

About 10 mL of the NEs were placed in closed test tubes where they were observed periodically for 63 days. The samples were observed visually, checking for signs of instability, such as creaming and/or sedimentation, and the volume was measured with a ruler every 7 days. The creaming index is calculated according to Equation (1) [41]:

CI(%) = H_c_/H_t_ × 100 (1)

where CI = creaming index, H_c_ = creaming height and H_t_ = total emulsion height.

Viscosity was measured manually by depositing an aliquot of the formulation in an Ostwald viscometer and timing the flow time obtained at a diluted concentration in distilled water, according to the methodology adapted from Almeida et al. (1995) [42]. The measurements were made in triplicate. The specific viscosity was calculated using Equation (2):

η_sp_ = (t − t_0_)/t_0_ (2)

where η_sp_ = specific viscosity, t = flow time of the solution in the viscometer and t_0_ = flow time of the pure solvent in the viscometer.

### 4.7. Characterization of the NEs

Optical microscopy was carried out using an inverted trinocular optical microscope (ANATOMIC). The micrographs of the NEs in an aqueous medium were captured using a 400× objective and processed using ImageJ software version 1.53t. The morphological characterization of the dried film nanoemulsions was conducted using SEM in Microscope Quanta 450-FEG equipment (FEI) (Taoyuan City, Taiwan) at 20 kV and 100,000× magnification. The films were pre-coated with gold using a cathodic spray applicator. The particle size and zeta potential measurements were carried out on a Malvern Zetasizer/Nanoseries ZS90 (Malvern, UK). The samples were diluted in distilled water with a ratio of 1:100 and kept under continuous stirring for 24 h before reading. The measurements were taken in triplicate.

### 4.8. Cytotoxicity

Cell toxicity was quantified by the ability of living cells to reduce the yellow dye 3-(4,5-dimethyl-2-thiozolyl)-2,5-diphenyl-2H-tetrazolium bromide (MTT) to a purple formazan product [43]. For the experiments, human leukocytes were plated in 96-well plates (0.7 × 10^5^ cells/well). The tested compounds (0.46 to 120 μg/mL) dissolved in DMSO were added to each well and incubated for 24 h. Bleomycin (0.39–100 μg/mL) were used as a positive control. Thereafter, the plates were centrifuged and fresh medium (150 μL) containing MTT at 0.5 mg/mL was placed in each well. Three hours later, the formazan product was dissolved in 150 µL of DMSO, and absorbance was measured using a multiplate reader (Spectra Count, Packard, ON, Canada). The drug effect was quantified as the percentage of absorbance of the reduced dye at 595 nm. Three repetitions were carried out for the tests.

### 4.9. Hemolytic Assay

The hemolytic test was performed in 96-well plates following the method described by Berlinck et al. [44] with a few modifications. Each well received 50 µL of 0.85% NaCl solution containing 10 mM CaCl_2_. The first well was the negative control (3% Tween). In the second well, 50 µL of the tested compounds (0.5 mg/mL) and 50 µL of Tween (3%) were added. The tested compounds were tested at concentrations ranging from 0.78 to 200 µg/mL. The compounds were serially diluted with 0.85% NaCl until the 11th well in a row. The last well received 50 µL of 0.2% Triton X-100 (in saline) to obtain 100% hemolysis (positive control). Each well then received 100 µL of a 2% suspension of human erythrocytes in saline containing 10 mM CaCl_2_. After incubation at room temperature for 1 h and centrifugation, the supernatants from the wells were transferred to a new plate and the hemoglobin released was measured using a multiplate reader (Spectra Count, Packard, ON, Canada) at 540 nm. The experiments were performed in triplicate in three independent trials. The methodology applied in the present study was approved by the Human Research Ethics Committee at the Federal University of Ceará (COMEPE-UFC–protocol number 281/09) and are in accordance with Brazilian research guidelines (Law 466/2012, National Council of Health) and with the Declaration of Helsinki.

### 4.10. Encapsulation Efficiency (EE%) and In Vitro Release Kinetics

The emulsions were previously diluted in a 1:4 (*v*/*v*) ratio of NE in 96% ethyl alcohol. The mixtures were left to stand for at least 24 h. Their absorbances were measured in a KASUAKI UV-VIS spectrophotometer. The concentration of chalcone (DB4OCH_3_) in the NE will be determined using the calibration curve of the sample from a 500 ppm stock solution in 96% ethyl alcohol, as a function of the absorbance values, according to Equation (3). The absorbances of the respective dilutions were determined on the spectrophotometer at the maximum wavelength of DB4OCH_3_ at 350 nm. The measurements were made in triplicate.

*y* = 0.0876 + 0.0001 r^2^ = 0.9975 (3)

The EE% of the NEs was calculated using Equation (4):

EE% = C_total_ + C_free_/C_total_ × 100 (4)

where C_total_ = the initial concentration of the compound added to NE and C_free_ = the calculated concentration of (DB4OCH_3_) in the formulation.

For the release kinetics, 300 mg of NE and 10 mL of a pH 7.0 phosphate buffer solution were added to a Falcon tube. The Falcon is closed using a dialysis membrane, which is immersed in a beaker containing 200 mL of a pH 7 buffer solution. The release system is kept under magnetic stirring at a constant temperature of 25 °C. At specific time intervals, aliquots of 3.0 mL are removed from the system, allowing analysis of the chalcone content released by the NE, after which the aliquot is returned to the system and read in a UV-VIS spectrophotometer. In order to elucidate the drug release mechanism, kinetic mathematical models were used. The data from the in vitro release curves were linearized according to zero-order and first-order kinetic models. This approach allowed for a more in-depth analysis of the points obtained, contributing to an understanding of the drug release process.

### 4.11. Antifungal Susceptibility Against Candida albicans

For antifungal activity, clinical strains of *C. albicans* (ATCC (90028), LABMIC (0102), LABMIC (0104), LABMIC (0105) and LABMIC (0128) were used. The minimum inhibitory concentration (MIC) for the *C. albicans* strains was determined using the broth microdilution method according to the methodology described by Fontenelle et al. [45], with the standards of the Clinical Laboratory Standards Institute protocol-M38-A [46].

For this test, 96-well microdilution plates were used, where 50 μL of RPMI medium was initially added to all the wells, followed by 50 μL of the sample. The sample was added to all the wells in the first column, followed by serial dilutions. A serial dilution covering the concentration range of 0.0024–2.50 and 0.0012–1.25 mg/mL in RPMI 1640 medium with L-glutamine (pH 7.4) was prepared from DB4OCH_3_ and NE stock solution, respectively. Amphotericin B was used as a standard drug control in the range of 0.015–16 μg/mL.

Finally, 50 μL of the inoculum was added to the plate wells. The plates were incubated at 37 °C and the visual reading was taken after 48 h for the *C. albicans* strains. All the tests were carried out in triplicate and the MIC was defined as the lowest concentration of the sample capable of inhibiting 100% of the visible growth of the microorganism. The results were determined by visualization as recommended by CLSI.

### 4.12. Molecular Docking Simulations

In the ligand preparation stage, DB4OCH_3_ was plotted and rendered in the Avogadro2 software version 1.99.0 (https://two.avogadro.cc/), which was configured to perform a structural optimization by the Merk Molecular Force Field (MMFF94) method at a convergence limit equal to 10⁻⁶ units using the Steepest Descent optimization algorithm.

The chemical structure of the enzymes Thymidylate Kinase (TMK) and DNA Gyrase B were taken from the RCSB Protein Data Bank (https://www.rcsb.org/), deposited under PDB codes 4QGG, 6F86 and 4HZ5, respectively, with expression systems in the Escherichia coli organism and chemical structures solved by X-ray diffraction at resolutions of 1.62, 1.90 and 2.70 Å, respectively. The enzymes were selected for their potential to regulate the topological state of bacterial DNA during replication [47].

The protein preparation stage includes the removal of water residues (H_2_O) and the addition of polar hydrogens and Gasteiger charges, using the AutoDockToolsTM program version 1.5.7 (https://autodock.scripps.edu/). The grid-box was delimited to cover the entire conformational space of the proteins, with dimensions x = 48 Å, y = 50 Å and z = 40 Å under axes x = 9.397, y = −6.887 and z = 2. 104 for the MTK enzyme, dimensions x = 126 Å, y = 126 Å and z = 126 Å under the coordinates x = 67.364, y = 30.317 and z = 54.751 for the DNA Gyrase B, and dimensions x = 50 Å, y = 54 Å and z = 52 Å under the axes x = −14.733, y = 14.965 and z = −7.09.

Thus, the AutoDockVinaTM code (https://vina.scripps.edu/) was configured to carry out a multiligand-based screening [48], using the Lamarckian genetic algorithm (LGA), with the best-pose selection criterion being the alignment between an affinity energy (EA) of less than −6.0 kcal/mol within a favorable statistical limit defined by a root mean square deviation (RMSD) of less than 2.0 Å [49].

### 4.13. MPO-Based DMPK Prediction

The two-dimensional chemical structure of DB4OCH_3_ was plotted and rendered using MarvinSketch^®^ Neon.3 LTS software, Chemaxon© (https://chemaxon.com/marvin). Quantitative druglikeness estimation was performed using the Multiparameter Optimization (MPO) algorithm from Pfizer, Inc., [50] as shown in Equation (5):

(5) $$MPO=\sum_{i=1}^{N}w_{k}T_{k}\left({x^{0}}_{k}\right)$$

where *w* represents the weighting factor (ranging from 0 to 1) assigned to each physicochemical property (*k*) in the linear and bilateral functions (*T*(*x*)) formed by the thresholds of intrinsic lipophilicity (logP) ≤ 3, buffer lipophilicity (logD at pH 7.4) ≤ 2, molecular weight (MW) ≤ 360 g/mol, Topological Polar Surface Area (TPSA) of 40–90 Å^2^, H-bond donors (HBD) ≤ 1 and basic pKa ≤ 8.0 (N = 6). The results were related to the molecular lipophilicity potential (MLP) surface map for the relationship between lipophilicity and polarity descriptors, using Python Molecular Graphics (PyMOL) version 2.x from the Schrödinger^®^ program [51].

To corroborate the pharmacokinetic profile observed in the MPO analyses, the drug metabolism and pharmacokinetics (DMPK) properties were predicted by the consensus predictive test between the ADMETlab 3.0 (https://admetlab3.scbdd.com/), Deep-PK (https://biosig.lab.uq.edu.au/deeppk/), ADMET-AI (https://admet.ai.greenstonebio.com/) and ADMET–LMC (http://qsar.chem.msu.ru/admet/), which include Parallel Artificial Membrane Permeability Assay (PAMPA) descriptors expressed in passive cell permeability (Papp, A → B) in colorectal adenocarcinoma (Caco-2) and Madin–Darby Canine Kidney (MDCK) cell models, P-glycoprotein (P-gp) inhibition and liver clearance rate (CLMicro) [52].

The prediction of the organic toxicity profile was based on the structural similarity test with reactive molecular fragments, based on the machine learning model of the predictive tools SMARTCyp (https://smartcyp.sund.ku.dk/), STopTox (https://stoptox.mml.unc.edu/) and Pred-hERG (http://predherg.labmol.com.br/), to identify metabolism sites dependent on cytochrome P450 (CYP450) isoforms and human ether-a-go-go-related gene (hERG) inhibitor molecular fragments, related to Drug-Induced Liver Injury (DILI) and cardiotoxicity endpoints, respectively.

## 5. Conclusions

A comparison of the homogenization techniques revealed that high-speed homogenization combined with the ultrasound technique produced nanoemulsions enriched with the chalcone DB4OCH_3_ that were more effective both in inhibiting the fungus *C. albicans* with a minimum inhibitory concentration with excellent results compared to existing literature and in terms of encapsulation efficiency and controlled release and other physicochemical characterizations. These results are significant as they highlight the ability of nanoemulsions to encapsulate and protect DB4OCH_3_, optimizing its bioactive properties. The molecular docking simulations showed that the aromatic nature of DB4OCH_3_ resulted in the formation of apolar interactions with aromatic residues located in the active site of the TMK, similar to the process that occurs in their respective co-crystallized inhibitors, within an affinity energy range that enables optimum specificity of the ligand for these two pathways. In addition, the predictive pharmacokinetic analyses showed that the compound has a high passive cell permeability and a low hepatic clearance, although phase I metabolism reduces its oral bioavailability and reduces its metabolic stability, suggesting a promising active ingredient as an oral drug with control of the daily oral dose administered. This has promising implications for the development of more effective antifungal therapies.

## Figures and Tables

**Table 1:** Particle size (PZ), zeta potential (ZP), polydispersity index (PdI) and encapsulation efficiency.

Code	Concentration (mg/mL)	PZ (nm)	PdI	ZP (mV)	EE (%)
UHS-7	0.07	195.70 ± 2.69	0.525 ± 0.01	−83.97 ± 5.01	92.10 ± 0.77
UHS-12	1.20	218.60 ± 3.11	0.492 ± 0.01	−88.83 ± 5.14	91.70 ± 1.15
U-7	0.07	196.30 ± 1.79	0.518 ± 0.01	−76.90 ± 4.44	39.40 ± 0.10
U-12	1.20	243.40 ± 4.49	0.509 ± 0.01	−91.77 ± 5.58	74.00 ± 0.28

**Table 2:** Minimum inhibitory concentration (MIC) against *Candida albicans*.

	*C. albicans* Strains				
Samples	ATCC (90028)	LABMIC (0102)	LABMIC (0104)	LABMIC (0105)	LABMIC (0128)
	CIM (µg/mL)				
CONTROL	N.I	N.I	N.I	N.I	N.I
DB4OCH_3_ (10.000 µg/mL)	312	312	625	625	625
UHS-7 (70 µg/mL)	17.5	17.5	8.75	17.5	8.75
UHS-12 (1.200 µg/mL)	75	75	75	37.5	75
ANF. B (16 µg/mL)	1	1	1	1	1

**Table 3:** Cytotoxicity in human leukocytes and hemolytic results in human erythrocytes of DB4OCH_3_ free, UHS-7, UHS-12, U-7, U-12.

Samples	Cytotoxicity ¹	Hemolytic Assay ^2^
	IC_50_ (µg/mL) ± S.E.M.	EC_50_ (µg/mL) ± S.E.M.
DB4OCH_3_	>150	>200
UHS-7	45.61 ± 1.73	>200
UHS-12	107.52 ± 2.56	>200
U-7	38.45 ± 1.10	>200
U-12	83.90 ± 5.10	>200
Bleomycin	30.48 ± 3.55	-

^1,2^ average values obtained from n = 3 measurements.

**Table 4:** Kinetic constant (K) and the correlation coefficient (R^2^) for the kinetic models studied: zero order, first-order.

NE	Zero-Order		First-Order		Higuchi		Hixson–Crowell		Korsmeyer–Peppas	
	R^2^	K_0_ (h^−1^)	R^2^	K_t_ (h^−1^)	R^2^	K_H_ (h^−1/2^)	R^2^	K_HC_ (mg/h^1/2^)	R^2^	K_k_ (h^−n^)
DB4OCH_3_	0.9511	0.0021	0.9102	0.0005	0.8944	0.1089	0.9370	0.0005	0.8269	0.6701
UHS-7	0.9542	0.0364	0.9564	0.0004	0.9192	1.9371	0.9619	0.0011	0.8835	0.532

**Table 5:** Data from molecular docking simulations expressed as RMSD and affinity energy (EA) and details of ligand–receptor interactions expressed as interaction type, residue and interaction distance.

Target	Ligand	RMSD (Å)	E_A_ (kcal/mol)	Interactions		
				Type	Residue	Distance (Å)
TMK	DB4OCH_3_	0.584	−6.767	Hydrophobic	Tyr100	3.53
					Tyr100	3.62
					Phe159	3.45
				H-bond	Arg70	2.86
				π-Stacking	Phe66	4.10
					Phe159	5.00
	OXA *	1.273	−7.233	Hydrophobic	Tyr100	3.75
				H-bond	Phe66	3.99
					Tyr100	4.32
				Salt Bridges	Lys15	3.68
					Arg92	5.47
	32C **	1.95	−7.67	Hydrophobic	Arg48	3.65
					Leu52	3.94
					Arg92	3.77
					Tyr100	3.82
				H-bond	Arg70	3.16
					Arg70	1.89
					Gln101	1.95
					Gln101	1.78
				π-Stacking	Phe66	3.87
					Phe66	4.80
				Salt Bridges	Arg48	3.48
Gyrase B	DB4OCH_3_	0.563	−6.101	Hydrophobic	Ile78	3.58
					Ile94	3.42
					Ile94	3.82
				H-bond	Val120	3.33
					Ser121	2.63
					Thr165	2.94
	OXA *	1.987	−6.857	Hydrophobic	Asn46	3.65
					Glu50	3.62
					Ile78	3.41
					Thr165	3.82
				H-bond	Glu50	2.91
				Salt bridge	Arg136	3.53
	CWW **	2.927	−4.381	Hydrophobic	Val43	3.83
					Val71	3.86
					Ile78	3.54
				H-bond	Asn46	2.83
					Asp73	1.89
					Asp73	1.92
					Arg76	3.58
					Gly77	1.81
					Thr165	3.09
				Water bridge	Gly77	2.70

Note: * Known drug used as a comparative in the molecular docking simulations; ** Inhibitor co-crystallized to the target.

**Table 6:** Physicochemical properties of DB4OCH_3_ calculated and applied to the Pfizer, Inc., classification criteria.

Property	Value	T0
logP	3.36	0.82
logD_7.4_	3.39	0.30
TPSA	35.53 Å^2^	0.77
MW	294.13 g/mol	1.00
HBD	0	1.00
pKa basic	2.81	1.00
MPO score	4.90	
Pfizer rule	Alert: (logP > 3 and TPSA < 75 Å^2^)	

**Table 7:** Drug metabolism and pharmacokinetics (DMPK) properties predicted for DB4OCH_3_ using the deep learning model-based tools ADMETlab 3.0 and Deep-PK.

Property	ADMETlab 3.0	Deep-PK
*P*_app,A→B_ Caco-2	1.12 × 10^−5^ cm/s	2.57 × 10^−5^ cm/s
*P*_app,A→B_ MDCK	2.16 × 10^−5^ cm/s	6.91 × 10^−5^ cm/s
P-gp inhibition	Inhibitor	Inhibitor
VDss	0.59 L/kg	1.87 L/kg
CYP2C19	Non-Substrate	Non-Substrate
CYP2D6	Substrate	Substrate
CYP3A4	Non-Substrate	Non-Substrate
CL_Micro_	11.48 mL/min/kg	5.95 mL/min/kg
DILI	0.82	Toxic

**Table 8:** Reaction characteristics of nanoemulsions.

Code	Concentration (mg/mL)	Technique
UHS-7	−	+
UHS-12	+	+
U-7	−	−
U-12	+	−

## Data Availability

Data is contained within the article.
